# Genome-wide identification of exon extension/shrinkage events induced by splice-site-creating mutations

**DOI:** 10.1080/15476286.2022.2139111

**Published:** 2022-11-03

**Authors:** Zhuo Qu, Narumi Sakaguchi, Chie Kikutake, Mikita Suyama

**Affiliations:** Division of Bioinformatics, Medical Institute of Bioregulation, Kyushu University, Fukuoka, Japan

**Keywords:** Splice-site-creating mutation, exon extension, exon shrinkage, RNA-Seq, personal genome

## Abstract

Mutations that affect phenotypes have been identified primarily as those that directly alter amino acid sequences or disrupt splice sites. However, some mutations not located in functionally important sites can also affect phenotypes, such as splice-site-creating mutations (SCMs). To investigate how frequent exon extension/shrinkage events induced by SCMs occur in normal individuals, we used personal genome sequencing data and transcriptome data of the corresponding individuals and identified 371 exon extension/shrinkage events in normal individuals. This number was about three times higher than the number of pseudo-exon activation events identified in the previous study. The average numbers of exon extension and exon shrinkage events in each sample were 3.3 and 11.2, respectively. We also evaluated the impact of exon extension/shrinkage events on the resulting transcripts and their protein products and found that 40.2% of the identified events may have possible functional impacts by either generating premature termination codons in transcripts or affecting protein domains. Our results indicated that a certain fraction of SCMs identified in this study can be pathogenic mutations by creating novel splice sites.

## Introduction

Mutations that affect phenotypes have been identified primarily as directly altering amino acid sequences or disrupting splice sites, i.e. disrupting some existing functions [1,2]. Exome sequencing is a method that efficiently identifies these mutations and has been successfully applied to identify causative mutations in various genetic disorders [3,4]. However, increasing studies have demonstrated that some mutations affecting the phenotype are not present in functionally important sites [5–7]. Splice-site-creating mutations (SCMs) are mutations that create a novel splice site at a locus not normally present, resulting in the generation of abnormal transcripts [8–11]. Such mutations can be located in deep intronic regions, triggering pseudo-exon activations and producing abnormal transcripts containing additional exons [12]. In addition, they can also be located near or within exons, causing exon extension or exon shrinkage events by creating novel splice sites, including those caused by synonymous mutations that may affect splicing [13,14].

The identification and analysis of abnormal splicing events caused by SCMs in the human genome have become necessary because these events can potentially cause genetic disorders. Indeed, some studies have systematically identified and analysed the impact of SCMs in disease samples [8,9,15]. It is also worth noting that a certain number of such mutations have been demonstrated even in healthy samples. Still, only pseudo-exon activations deep in intronic regions were analysed in the previous study [16], and those occurring near or within exons, which lead to exon extension/shrinkage events, have not been analysed so far.

In this study, we used personal genome sequencing data and transcriptome data of the corresponding individuals to identify SCMs that can cause exon extension/shrinkage events in healthy individuals. We performed a comparative analysis of previous studies of pseudo-exon activations [16] and found that exon extension/shrinkage-inducing SCMs occurred approximately three times higher in these healthy individuals. We also evaluated the impact of SCMs on the resulting transcripts and their protein products. Our results indicated that a certain fraction of SCMs identified in this study can be pathogenic mutations by creating novel splice sites.

## Materials and methods

### Genomic variation and transcriptome data

We used individual genomic variation data in the Variation Call Format (VCF) from the 1000 Genomes Project [17]. These have already been registered as single-nucleotide polymorphisms (SNPs) in dbSNP [18]. We used transcriptome data from lymphoblastoid cell lines of 462 corresponding individuals from the GEUVADIS project [19]. As in the previous study [16], we retained the transcriptome data of 235 high-quality individuals with sequencing quality scores of >30 as calculated by the FastQC program (http://www.bioinformatics.babraham.ac.uk/projects/fastqc) for further processing.

### Construction of individual-specific reference genome and RNA sequencing (RNA-seq) read mapping

We used reference genome sequence data for the hg19 version and reference transcriptome data documenting the gene structure in General Transfer Format (GTF) format from the UCSC Genome Browser [20]. BCFtools (version 1.9) [21] was used to construct 235 individual-specific reference genome sequences based on the reference genome sequence and the variation information of each individual. HISAT2 (version 2.1.0) [22] was used to map the RNA-seq data of each individual to the individual-specific genome sequence of the corresponding individual, reflecting individual variation information. The program’s default parameters were used for mapping.

### Data visualization

We used the Integrative Genomics Viewer (IGV) software (version 2.9.4) [23] to visualize the mapping, individual variant, and gene structure data. WebLog3 [24] (http://weblogo.threeplusone.com/) was used to visualize the base frequencies at splice sites.

### Splice site scoring

The splice site strength was assessed using the MaxEntScan [25] and SpliceAI (version 1.3) [26] programs. In the MaxEntScan program, the strength of each site as a splice site can be predicted based on the sequence. For donor splice site, genome sequence segments corresponding to the three bases at the end of the extended/shrunken exons and the six bases at the start of the introns were subjected; for acceptor splice site, genome sequence segments corresponding to the 20 bases at the end of introns and the 3 bases at the start of the extended/shrunken exons were subjected.

### Calculation of junction allele fraction

The junction allele fraction (JAF) was defined as the ratio of the number of junction reads supporting certain splicing relative to the total junction reads and is used to measure the relative amount of the extended/shrunken exon–intron junctions created by SCMs compared to annotation junctions [8]. JAF can be calculated using the following equation:

JAF = *J*n/(*J*n + *J*a),

where *J*n is the number of junction reads supporting the novel splice site and *J*a is the number of junction reads supporting the annotated splice site. The JAF value ranges from 0 (when entirely using annotated exons) to 1 (when entirely using extended/shrunken exons).

### Analysis of protein domain structure and gene function

In cases where the activation of exon extension/shrinkage did not generate premature termination codons (PTCs), we used the hmmscan program in HMMER (version 3.3.2) [27] to examine the effect of the presence of extended/shrunken exon on protein domains. The tool can be run from the command line, and the input data were the amino acid sequences derived from the mRNA containing the exon in which extension/shrinkage events happened. The E-value of 0.0001 was used as the threshold to judge whether the input amino acid sequence contains a known protein domain registered in the protein profile database. The possible association between SCMs and disease pathogenesis was analysed by evaluating whether the identified SCMs were located in the causative gene of genetic disease (mainly Mendelian disease). The list of causative genes of genetic diseases was obtained from the OMIM [28] (https://www.omim.org/). To see whether these SCMs had already been classified as pathogenic in prior studies, we checked if the variants were registered in the ClinVar [29] database.

### Enrichment analysis of functional categories of genes

We used Metascape [30] for enrichment analysis of functional categories of genes. The following settings were used: ‘Input as species’ was set to ‘H. sapiens’, and ‘Analysis as species’ was set to ‘H. sapiens’.

### Statistical analyses

Statistical analyses were performed using the R software (version 4.1.0). *t*-Test was used to determine whether the SpliceAI scores were significantly different between the identified SCMs and randomly selected 10,000 SNVs, and a *p*-value of <0.01 was considered statistically significant.

## Results

### Identification of exon extension and shrinkage events and the associated SCMs

**Figure 1.**
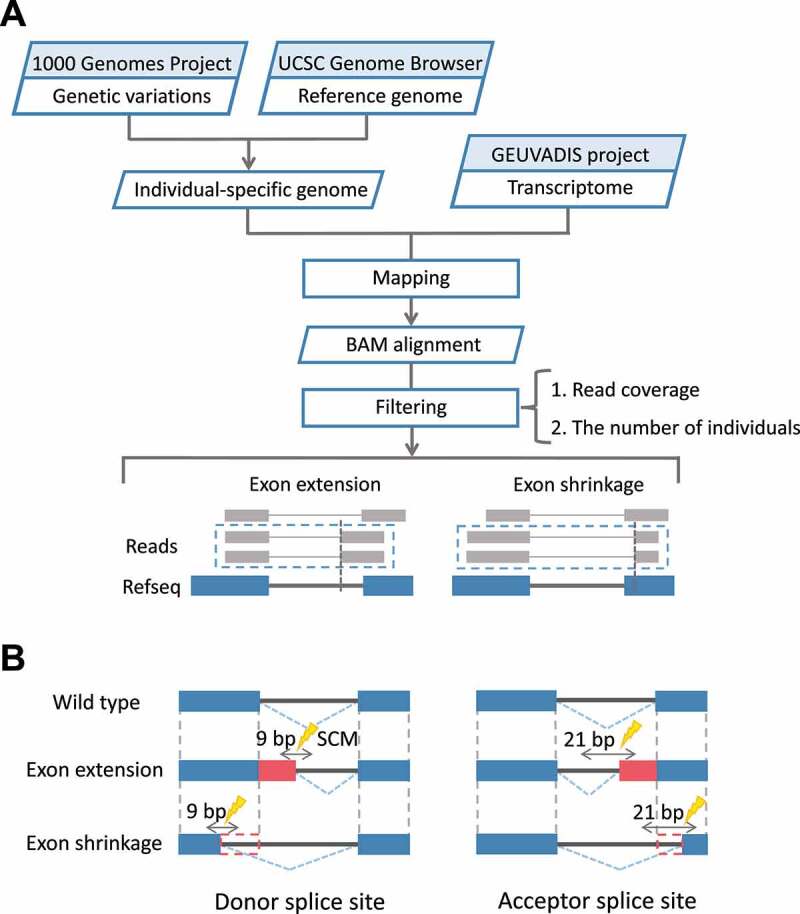
Workflow for the identification of SCMs causing exon extension/shrinkage events. (A) By constructing individual-specific genomes and mapping the transcriptome data of the corresponding individuals to the genomes, the obtained junction reads were used to identify exon extension/shrinkage events. (B) SNVs in the donor splice site or acceptor splice site regions of the extended/shrunken exons were identified as candidate SCMs. For the 3 bp at the end of the exon and the 6 bp at the start of the intron, a total of 9 bp was defined as the donor splice site; for the 18 bp at the end of the intron and the 3 bp at the start of the exon, a total of 21 bp was defined as the acceptor splice site.

Exon extension and shrinkage events were first identified through the following steps (Figure 1(a)). For this, we constructed individual-specific genomes that reflect the variation information of the individuals, as similar to the previous studies identifying pseudo-exon activation events [16]. This step was necessary because our purpose was to find exon extension/shrinkage events from junction reads obtained by mapping transcriptome data to the genome and then find the single-nucleotide variants (SNVs) that can cause these events. If the genuine reference genome was used for mapping an RNA-seq data for an individual, we might fail to correctly map junction reads at the site where an SCM is created by an SNV for that individual, making it difficult to identify exon extension/shrinkage events of each individual.

Next, we collected junction reads in which the mapping positions on one side were consistent with the positions of annotated exons in RefSeq transcripts, and the mapping positions on the other were inconsistent with the positions of the annotated exons. To reduce the error rate in the mapping process, only those junctions covered by two or more junction reads were further selected as candidates for exon extension/shrinkage events. We also applied the condition that, in these junction reads, the side for which the mapping position was inconsistent with the annotated exons must have at least 5 bp. For junctions satisfying the above conditions, the exon located at the side inconsistent with the annotated exon was defined as an extended or shrunken exon.

To identify candidate SCMs responsible for these events, we focused on SNVs located at the flanking region of donor splice site or acceptor splice site of these extended/shrunken exons (Figure 1(b)). Here, we considered 3 bases at the end of the extended/shrunken exons and 6 bases at the start of the intron as the flanking region of donor splice site and 18 bases at the end of the intron and 3 bases at the start of the extended/shrunken exons as the flanking region of acceptor splice site.

We further analysed the correspondence between SNVs and exon extension/shrinkage events across all individuals. Only when the number of individuals with both SNV and exon extension/shrinkage event was greater than the number of individuals with the same SNV but no corresponding event, the SNV was further judged as the candidate SCM. This condition was set because, for lowly expressed genes, we might not be able to identify exon extension/shrinkage events in some individuals, although they have SCM. Conversely, if a junction read was observed in an individual who does not have the SNV, such SNV was excluded from the candidate SCMs. This is because, in this case, it is evident that the SNV is not an SCM.

Through the above analysis of the 235 individuals, 371 exon extension/shrinkage events induced by the creation of novel splice sites by SCMs were identified in the total of 341 genes, including 128 extension events (37 at donor splice site and 91 at acceptor splice site; Supplemental Table S1) and 243 shrinkage events (134 at donor splice site and 109 at acceptor splice site; Supplemental Table S2).

### Examples of exon extension and shrinkage events

**Figure 2.**
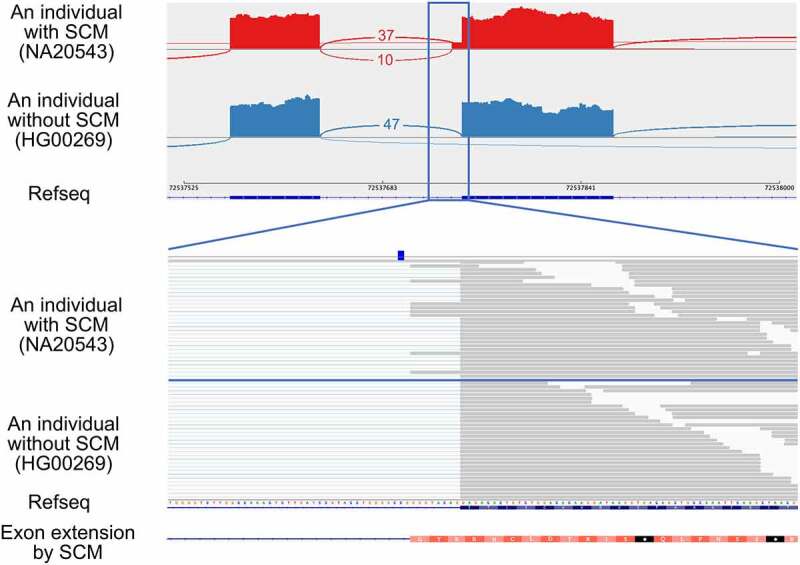
An example of an exon extension event identified in the exon 13 of *ATG16L2*, which was activated by the transversion of the SCM (rs142632291) from C-to-A so that the novel acceptor splice site was created. The upper panel shows the Sashimi plot of the extended exon and upstream exon observed in individuals with and without the SCM from top to bottom. Each number represents the number of exon–exon junction reads. Sample IDs are shown in parentheses. The lower panel shows a close-up view of the extended exon and the position of the SCM.

As an example, we here show an exon extension event identified at the acceptor splice site of the exon 13 (the 13th of 18) of autophagy-related 16 like 2 (*ATG16L2*) (Figure 2). The identified SCM is a C-to-A transversion that creates a canonical dinucleotide of acceptor splice site. We applied the MaxEntScan program, which predicts the strength of each splice site based on the sequence, to the sequences with and without the SCM to assess the potential to be an acceptor splice site. The MaxEntScan scores were 4.01 and −4.03, respectively, indicating that the sequence with the SCM had a much higher potential to be an acceptor splice site than the reference sequence at the corresponding region. We also applied SpliceAI to calculate whether the SCM has the potential to create a splice site, and the results showed that the probability of gaining the acceptor splice site by the SCM was 0.58, indicating that the SpliceAI also supported the variant to be SCM. The extended exon is 8 bp longer than the wild-type exon, which is not a multiple of three, so the extension disrupts the coding potential of its downstream. In this case, PTCs were introduced in the extended exon. The JAF value, which represents the relative amount of the extended exon–intron junctions created by SCM compared to annotated exon–intron junctions (see Materials and methods), was 0.21. Two possible factors affect the JAF value. One is the degradation of transcripts containing PTC by nonsense-mediated mRNA decay (NMD) [31] and the other factor is the difference in the splice site strength between the extended and annotated exons. The allele frequency of this mutation in the gnomAD database [32] was 0.0031, and there were three other individuals having this mutation in the samples analysed in this study. We confirmed that the same exon extension was also observed in these three individuals (Supplemental Fig. S1).

**Figure 3.**
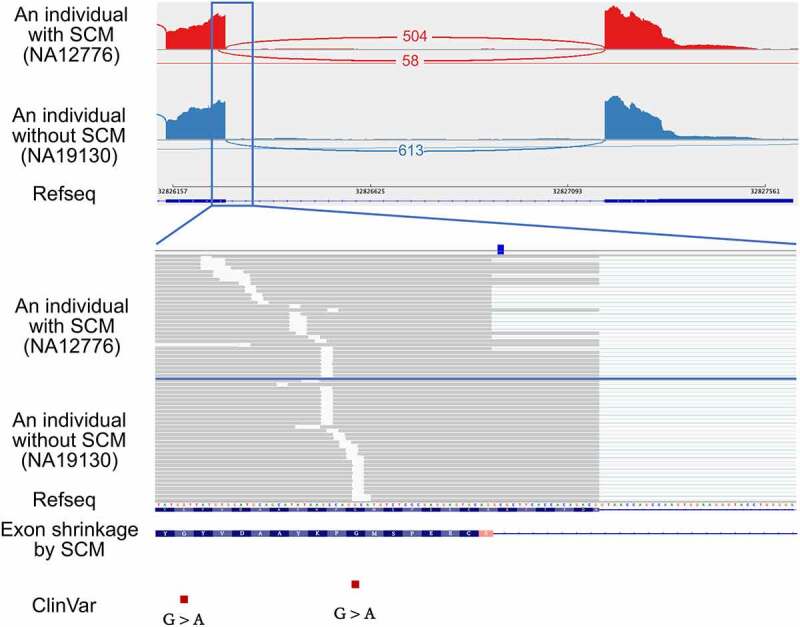
An example of an exon shrinkage event identified in the exon 5 of *PSMB9*, which was activated by the transition of the SCM (rs17213861) from C-to-T so that the novel donor splice site was created. The upper panel shows the Sashimi plot of the shrunken exon and downstream exon observed in individuals with and without the SCM from top to bottom. Each number represents the number of exon-exon junction reads. Sample IDs are shown in parentheses. The lower panel shows a close-up view of the shrunken exon and the position of the SCM. The red squares at the bottom represent pathogenic variants reported in ClinVar.

Another example is an exon shrinkage event identified at the donor splice site of the exon 5 (the fifth of 6) of proteasome 20S subunit beta 9 (*PSMB9*) (Figure 3). The identified SCM is a C-to-T transition that creates a canonical dinucleotide of donor splice site. Based on the donor splice site sequence of the shrunken exon, the MaxEntScan scores of the sequence at the donor splice site of the shrunken exon with and without the SCM were 4.83 and −2.93, respectively, indicating that the splice site strength was increased by the variant. The probability of gaining a donor splice site by the SCM was 0.43 using SpliceAI. The shrunken exon is 17 bp shorter than the wild-type exon, changing the intron phase from phase 1 to phase 2, resulting in a frameshift and generation of a PTC in the downstream exon. The JAF value for this case was 0.10. The allele frequency of this mutation in the gnomAD database [32] was 0.0033, and there were two other individuals having this mutation in the samples analysed in this study. We confirmed that the same exon shrinkage was also observed in these two individuals (Supplemental Fig. S2). Because *PSMB9*, which codes for proteasome subunit β1i, has already been identified as a causative gene for proteasome-associated autoinflammatory syndrome (PRAAS) by reduced activity or impaired function of the proteasome [33], we evaluated if the SCM can be a causative mutation. Although this variant has not been reported in ClinVar, the SCM might be highly damaging because of the PTC resulting from frameshift caused by the SCM-induced exon shrinkage. Even in individuals with SCM, the expression of this gene was not reduced, meaning that the transcript with PTC will lead to the production of truncated protein. Notably, two pathogenic variants have been reported in ClinVar at the close upstream of this SCM, further supporting the possibility of the SCM as a novel pathogenic variant.

### Characteristics of the identified SCMs

**Figure 4.**
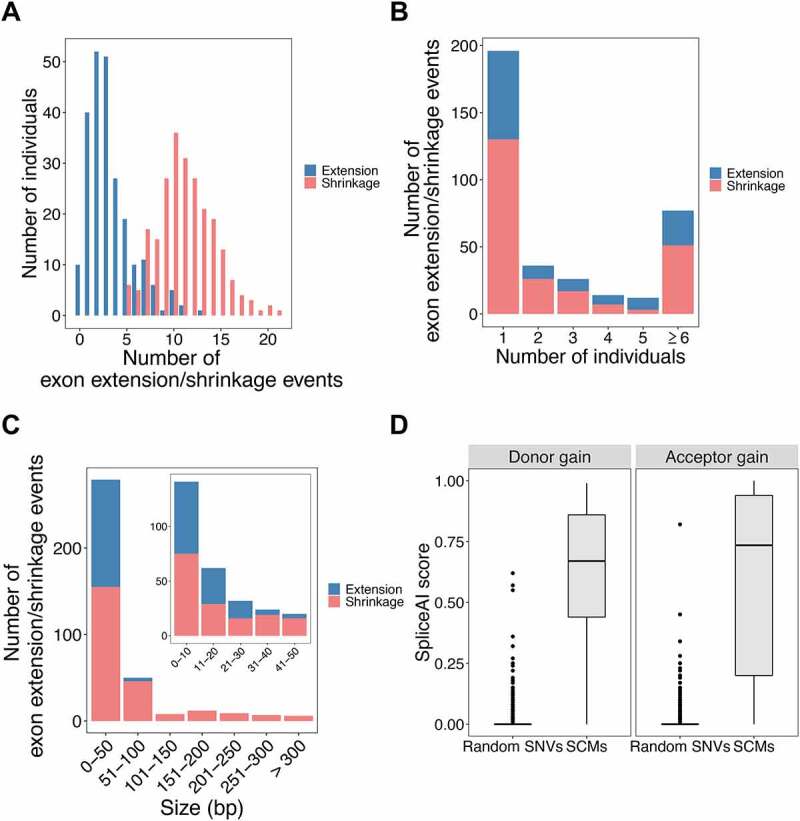
Basic characteristics of the identified exon extension/shrinkage events. (A) Histogram of the number of exon extension/shrinkage events per individual. (B) Histogram of the number of exon extension/shrinkage events shared among multiple individuals. (C) Histogram of the distribution of exon length changes due to extension/shrinkage events. (D) Boxplots of SpliceAI scores for SCMs and random SNVs, where SCMs were divided into acceptor and donor gain groups according to their locations.

Among the 235 individuals analysed in this study, the average numbers of exon extension and exon shrinkage events in each sample were 3.3 and 11.2, respectively. The maximum number of exon extension events found in an individual was 13, and this case was found in one individual. For exon shrinkage, the maximum number of events found in an individual was 21, which was also found in only one individual (Figure 4(a)). In addition, exon extension was not found in 10 individuals, whereas, for exon shrinkage, at least five events occurred in each individual. The identified exon extension/shrinkage events can be shared by different individuals. The maximum numbers of individuals sharing the same event were 99 and 209 for exon extension and shrinkage, respectively. Most events were observed in only one individual (67 exon extension events and 135 exon shrinkage events) (Figure 4(b)). Most events were observed in only one individual, probably due to the lower allele frequency (AF) of the associated SCMs. To confirm this, by analysing the relationship between the AF of the associated SCMs and the number of shared individuals, as expected, the AF of SCMs tended to increase with the increasing number of individuals sharing the SCM-induced events (Supplemental Fig. S3). The distribution of the length changes for exon extension and shrinkage events was also calculated (Figure 4(c)) as well as the length distribution of the original exons and flanking introns (Supplemental Fig. S4). Among the identified exon extension events, there were only four events with an extended length of >50 bp, and the maximum exon length change was 69 bp. In contrast, among the identified exon shrinkage events, there were six events with an exon length change of >300 bp, and the maximum exon length change was observed in the last exon of USP31, for which the exon shrank up to 4,797 bp (the length of the original exon was 8,120 bp). However, the length changes of both events were most abundant in 0–10 bp, and the numbers of events were similar for extension (66 events) and shrinkage (75 events). The reason why we did not observe only a few extension events with greater length change might be the limitation of the read length.

To assess the potential of the identified SCMs to be splice sites, we calculated the SpliceAI scores of both the SCMs and randomly selected 10,000 SNVs. For both SCMs that might create donor and acceptor splice sites, the identified SCMs tended to have significantly higher scores than the randomly selected SNVs, indicating that the SCMs are more likely to create novel splice sites (*p*-value <0.01) (Figure 4(d)). Although there are four SNVs that have rather high SpliceAI scores (>0.50) in the randomly selected SNVs, we did not see any exon extension/shrinkage events in the RNA-seq data having these SNVs. In addition, we also used the MaxEntScan program to evaluate the strength of each local sequence segment as a splice site and found that 97.3% of the identified exon extension/shrinkage events showed that the mutated sequence had a higher score than the reference sequence (Supplemental Fig. S5). We further analysed whether JAF values correlated with differences in MaxEntScan scores and distances between the authentic splice sites and those activated by the SCMs. JAF value tends to be high with the higher MaxEntScan score at SCM (Pearson’s *r* = 0.503, *p* = 9.96 × 10^−9^) (Supplemental Fig. S6). However, JAF values were not correlated with the distance between the authentic splice sites and those activated by SCMs (Pearson’s *r* = −0.122, *p* = 0.195).

To assess the effect of exon extension/shrinkage on the transcripts in terms of their coding potential, we analysed whether each identified exon extension/shrinkage can change the original reading frame and introduce PTC or disrupt the existing protein domains. Of the identified 371 exon extension/shrinkage events, 244 were located in the coding regions, of which 122 events were expected to generate PTCs in either extended/shrunken exons (67 events) or downstream exons by inducing frameshift (55 events) and then may induce NMD (Supplemental Fig. S7). A shrunken exon itself can have a PTC if a shrinkage of the length not multiple of three occurs at the 5’ end of the exon and hence introduces a frameshift. It is worth noting that, even with this mRNA quality-control mechanism, transcripts with exon extension/shrinkage can still be identified, probably because the NMD process often does not degrade the transcripts with PTC completely. This is supported by the studies that reported that many transcripts that may trigger NMD can still be detected in transcriptome data [19,34]. In addition, for the remaining 122 events in the coding region, we used the hmmscan program in HUMMER to analyse their protein sequences to see whether the exon extension/shrinkage would affect the existing protein domain. We found that 27 of them were located within the known protein domains, suggesting that domains may be disrupted by these events (Supplemental Fig. S7).

To explore the possibility of SCMs as novel pathogenic variants for genetic disorders, we counted the number of SCMs located within genes known to be responsible for genetic disorders using the OMIM database and further counted those SCMs that might disrupt the transcripts or the protein domains among them. There were 84 SCMs in the pathogenic gene listed in OMIM, in which 35 SCMs have the potential to introduce PTC and 5 SCMs seem to disrupt the protein domains. Among these 40 SCMs (35 + 5), only one SCM has been reported in ClinVar as pathogenic, and the rest of the SCMs might be novel candidates for pathogenic variants based on this study.

We also analysed the known associated phenotypes of the identified SCMs by searching these mutations in the ClinVar database and found that most (85.7%) of them have not been registered in ClinVar (Supplemental Tables S1 and S2). Even for those registered in ClinVar, only one mutation is classified as ‘Pathogenic’, which was the one found in the pathogenic gene listed in OMIM. The mutation was in the *SLC7A7* gene and was shown to affect the RNA splicing process [35].

We further performed GO enrichment analysis on the 341 genes with the exon extension/shrinkage events, and the result showed that genes involved in the cell cycle and DNA repair were enriched in the gene set though this might be the characteristics of the lymphoblastoid samples. To clarify this point, we also performed GO enrichment analysis on randomly selected 350 genes with some expression (FPKM > 1.0) and found that those terms were also enriched in the randomly selected genes, indicating that the enrichment is not due to the exon extension/shrinkage events but by cell lines used in the analysis.

### Frequency and spectrum of SCMs for each site

**Figure 5.**
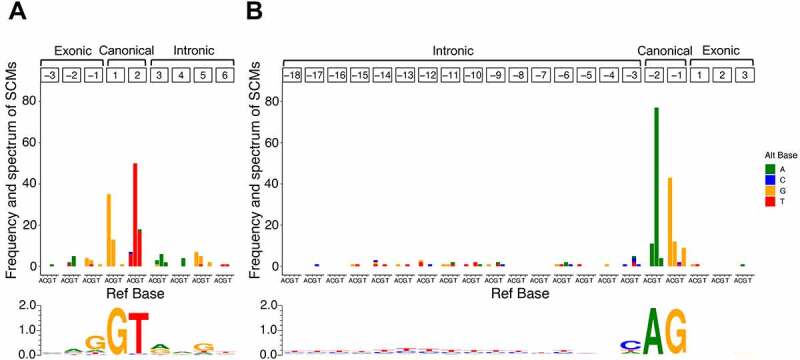
Frequency and spectrum of SCMs causing exon extension/shrinkage events. (A) Donor splice site and (B) acceptor splice site. The colour codes for alternative bases are shown on the right side of each panel. The base frequency data for splice sites of extended/shrunken exons were represented as a sequence logo using WebLogo 3. The positions of the SCMs are relative to the extended/shrunken exons.

For the identified SCMs, we summarized their positional frequency and spectrum relative to the exon–intron boundary newly created by the SCMs (Figure 5). As expected, most SCMs were observed in canonical dinucleotide at both donor splice site (Figure 5(a)) and acceptor splice site (Figure 5(b)). More specifically, in the donor splice site, 72.5% of the SCMs were observed in the canonical GU dinucleotide, and in the acceptor splice site, 79.0% of the SCMs were observed in the canonical AG dinucleotide. Also, most SCMs were substitutions that conform to splice site motifs [36].

**Figure 6.**
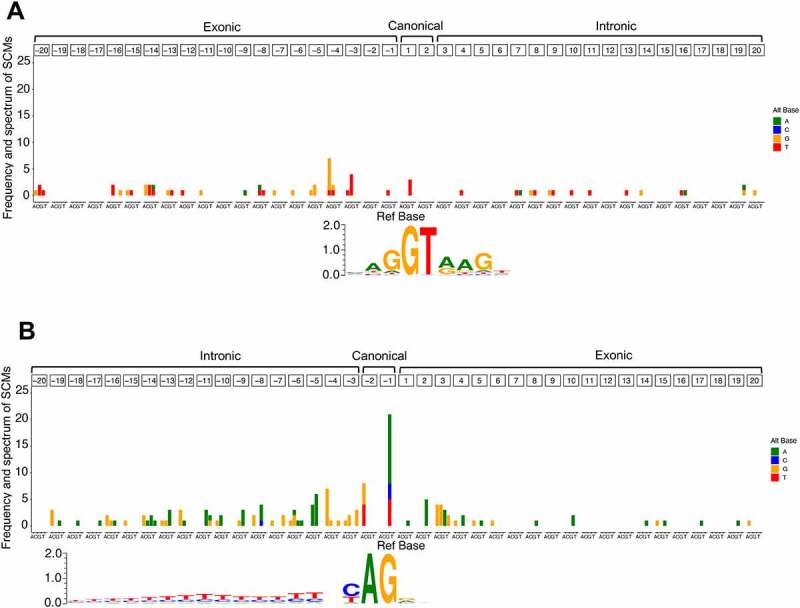
Frequency and spectrum of SCMs adjacent to annotated exons. (A) Donor splice site and (B) acceptor splice site. The colour codes for alternative bases are shown on the right side of each panel. The base frequency data for splice sites of all the annotated exons were represented as a sequence logo using WebLogo 3. The positions of the SCMs are relative to the annotated exons.

We also analysed the positional frequency and spectrum of the SCMs relative to the annotated original splice sites (Figure 6). In the donor splice site, SCMs showed no obvious positional preference and were relatively evenly distributed in exonic and intronic regions (Figure 6(a)), whereas, in the acceptor splice site, SCMs were rarely observed in exonic regions, except for the positions closer to the annotated original splice sites (Figure 6(b)). This may be because not only the canonical dinucleotide but also the existing polypyrimidine tracts play an important role in creating novel acceptor splice site. This might be supported by the fact that the number of SCMs gradually decreases with the distance from the annotated splice site. In addition, in the acceptor splice site, SCMs most commonly occur at the −1 position of the intronic region, which is the second base of the canonical dinucleotide. The SCMs were also relatively concentrated at the −5 and −4 positions of the intronic region, and the dominant alternate bases at these two positions were A and G, respectively. This trend may be related to the creation of the NAGNAG motif, which is often observed at acceptor splice site [37]. The dominant alternate bases of A and G observed at the 2 and 3 positions, respectively, of the exonic region might also be related to the creation of a novel NAGNAG motif.

## Discussion

In this study, we successfully identified 371 exon extension/shrinkage events in normal individuals and the SCMs that are thought to induce these events using individual-specific genomic data and the corresponding RNA-seq data. By constructing individual-specific genomic data that reflect the variation information for each individual, we were able to obtain more accurate junction reads representing splicing patterns from the RNA-seq data mapped onto the corresponding individual-specific genome. We analysed the effect of extended/shrunken exon due to SCMs on transcripts and proteins and found that the creation of novel splice sites introduced PTCs in transcripts or disrupted protein domains.

Of the 371 exon extension/shrinkage events identified, the number of exon shrinkage events was approximately 1.9 times greater than extension events. This difference might mainly be due to the limitation of the read length, which was further supported by the distribution of exon length changes in exon extension and shrinkage events. Among the identified extension events, the longest extended length was only 69 bp, compared to shrinkage events, which can be shrunken by >300 bp, whereas the changes in length were concentrated in 0–10 bp in both events, and the numbers of these two events were also similar. Because the read length of the RNA-seq data used in this study was only 75 bp, it was difficult to identify extension events with a change in length of >70 bp. However, in principle, it had no effect on the identification of exon shrinkage events. In addition, we found that the mutation spectrum of the identified SCMs and the sequence motifs of splice sites were highly consistent [36], further supporting the association of these SCMs with exon extension/shrinkage events.

Compared to the 116 pseudo-exon activation events identified in the previous study [16], we identified approximately three times as many exon extension/shrinkage events in this study. There are at least two possible reasons for the difference in the number of events. One reason might be that it is easier to create novel splice sites in exon extension/shrinkage events than in pseudo-exon activation events. This is because one side of the extended/shrunken exon uses the same annotated splice site, whereas, in pseudo-exon activation, not only the side where the SCM resides but also the other side of the pseudo-exon should have a cryptic splice site at the proper position. Another reason might be that there can be less impact on transcripts and proteins in the exon extension/shrinkage events. Indeed, among the identified extended/shrunken exons, the proportion of the cases that neither introduce PTC nor affect protein domains was approximately 25.6%, whereas the proportion of the above cases in pseudo-exon activation events identified in the previous study was approximately 12.9% [16]. One reason for this might be that, for example, in the formation of the NAGNAG motif inferred by the frequency and spectrum of SCMs, there is no PTC due to frameshift, and the effect on the protein is also minimal as it changes only one amino acid residue. Indeed, a total of 34 SCMs identified in this study (17.0% of SCMs found at acceptor sites) were found to form NAGNAG motifs.

The possibility of SCMs as causative variants of genetic disorders has often been overlooked. This is because they are often present in sites that have no defined function. To elucidate the extent to which the identified SCMs may be the cause of genetic disorders, we performed further analysis of the identified SCMs that were found in the genes known to cause genetic disorders. Among the SCMs identified in this study, 84 were located in the causative genes of genetic disorders, and 40 of them were thought to introduce PTCs or disrupt existing protein domains. Most of these variants were either not indexed by ClinVar, or if they were, they were not considered pathogenic. According to ClinVar, only one of these variants is reported to be pathogenic. Because the involvement of the rest of the variants in the cause of the disorders has not been established so far, those identified SCMs might be novel candidates as causative mutations. Our results demonstrated that when no pathogenic variants were found in coding regions or existing splice sites in identifying causative mutations for genetic disorders, it is worth considering the involvement of SCMs as causative mutations.

## Supplementary Material

Supplemental Material

Supplemental Material

## Data Availability

The data that support the findings of this study are available from the corresponding author, M.S., upon request.
