# Supplementary material for: Genome-wide identification of exon extension/shrinkage events induced by splice-site-creating mutations
Source: RNA Biol. 2022 Nov 3;19(1):1143–52. doi: 10.1080/15476286.2022.2139111 (PMC9639565; doi:10.1080/15476286.2022.2139111)
Supplement: Supplemental Material [file KRNB_A_2139111_SM1412.pdf]

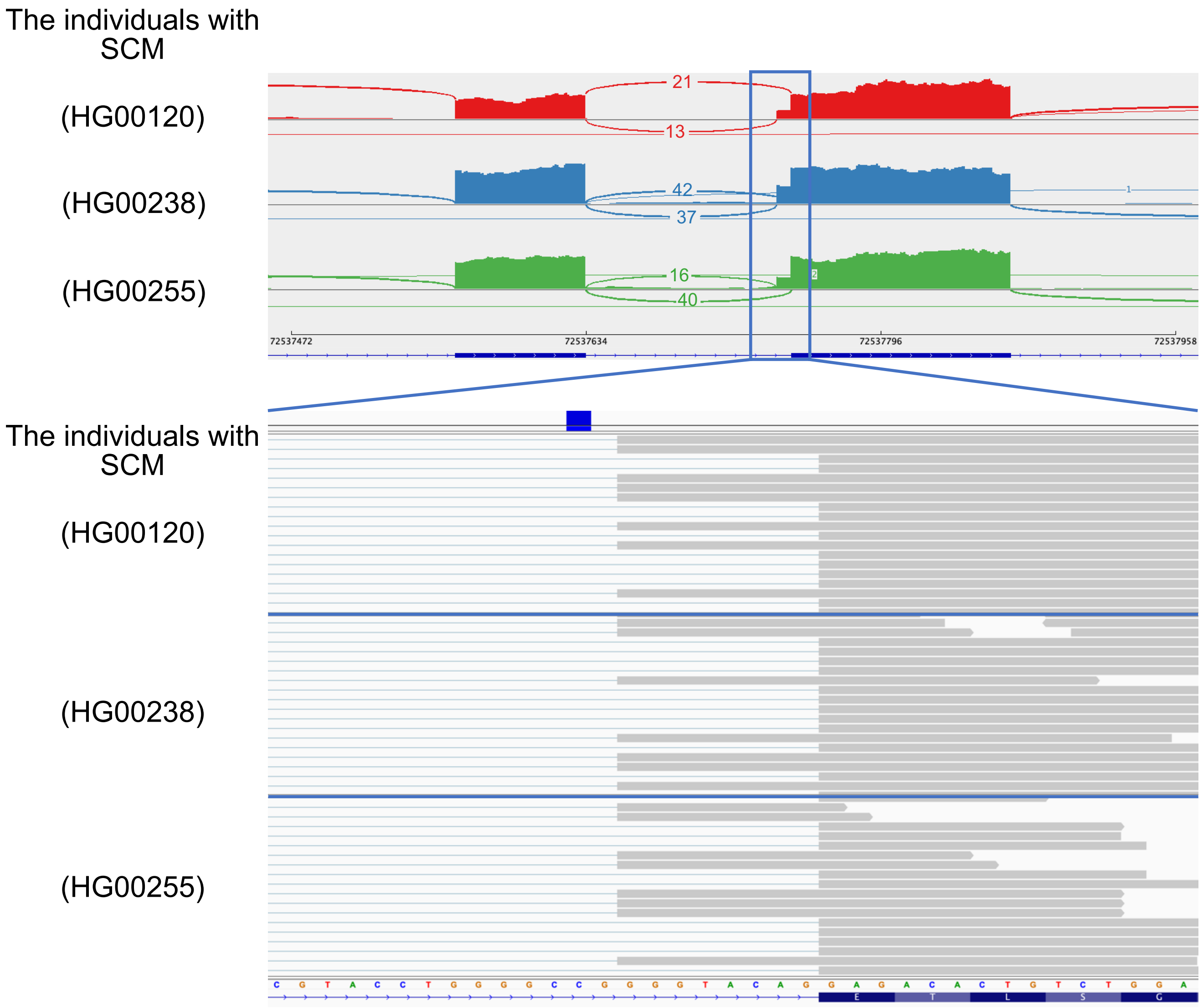

Supplemental Figure S1. Three other individuals with the SCM (rs142632291) which can activate an exon extension event in exon 13 of *ATG16L2*. The upper panel shows the Sashimi plot of the extended exon and upstream exon observed in the three individuals with the SCM. Each number represents the number of exon-exon junction reads. Sample IDs are shown in parentheses. The lower panel shows a close-up view of the extended exon and the position of the SCM.

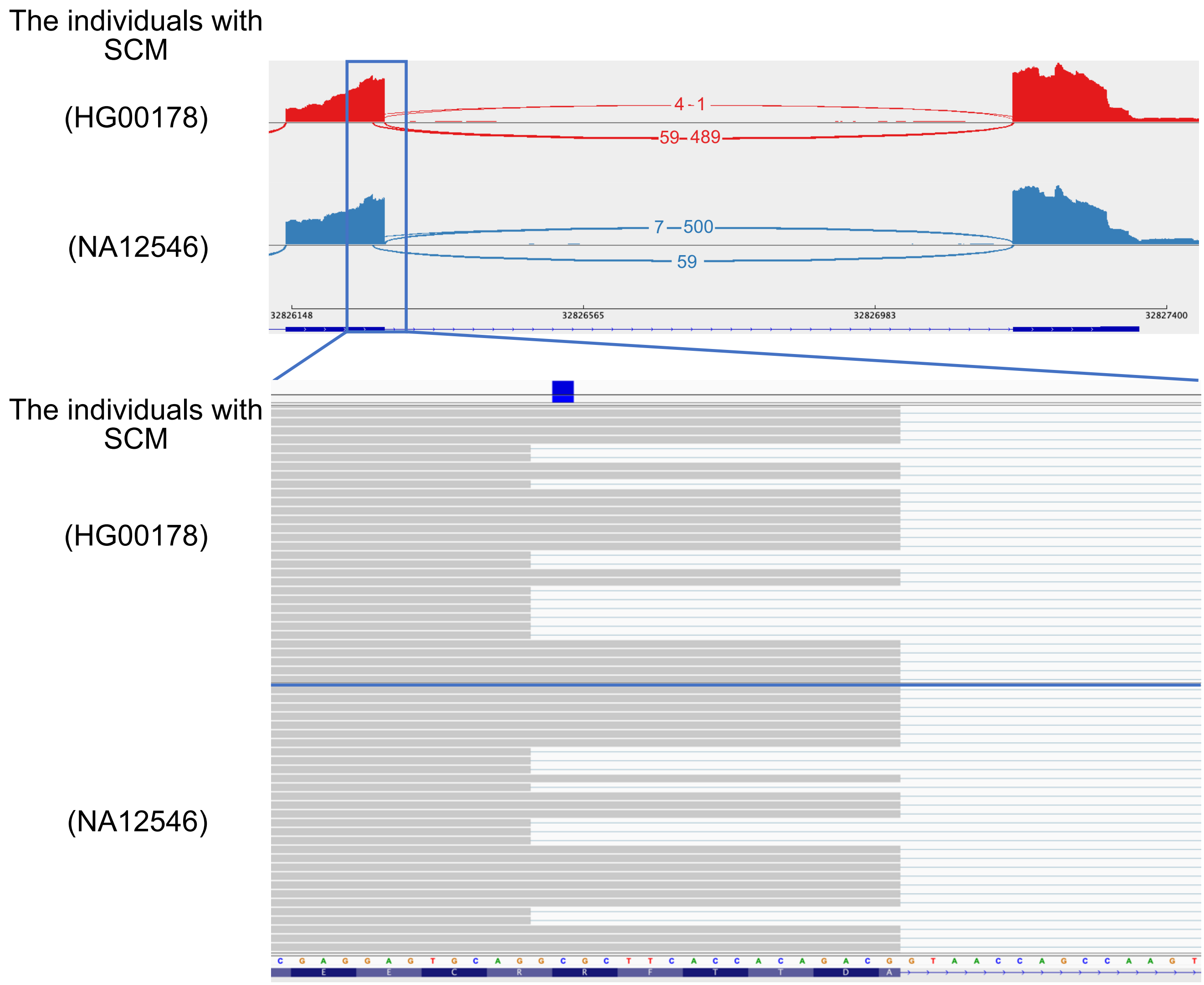

Supplemental Figure S2. Two other individuals with the SCM (rs17213861) which can activate an exon shrinkage event in exon 5 of *PSMB9*. The upper panel shows the Sashimi plot of the shrunken exon and downstream exon observed in the two individuals with the SCM. Each number represents the number of exon-exon junction reads. Sample IDs are shown in parentheses. The lower panel shows a close-up view of the shrunken exon and the position of the SCM.

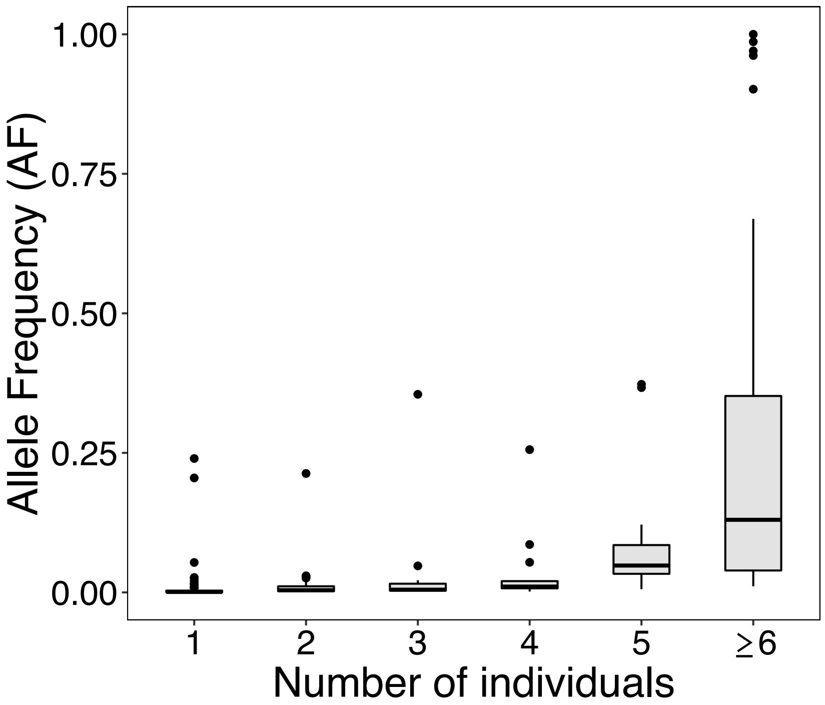

Supplemental Figure S3. Boxplots of allele frequencies of SCMs. Here the SCMs are divided into 6 groups according to the number of individuals they are shared with.

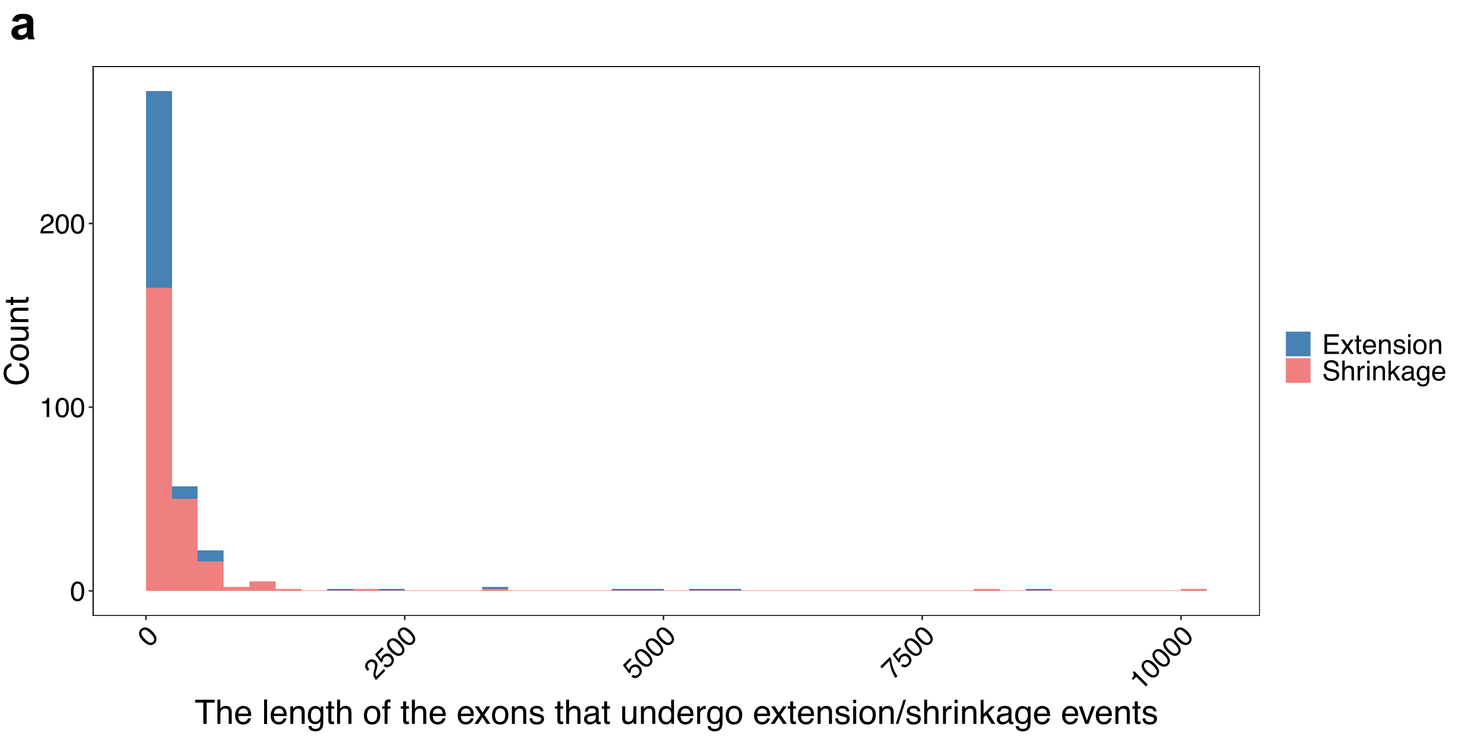

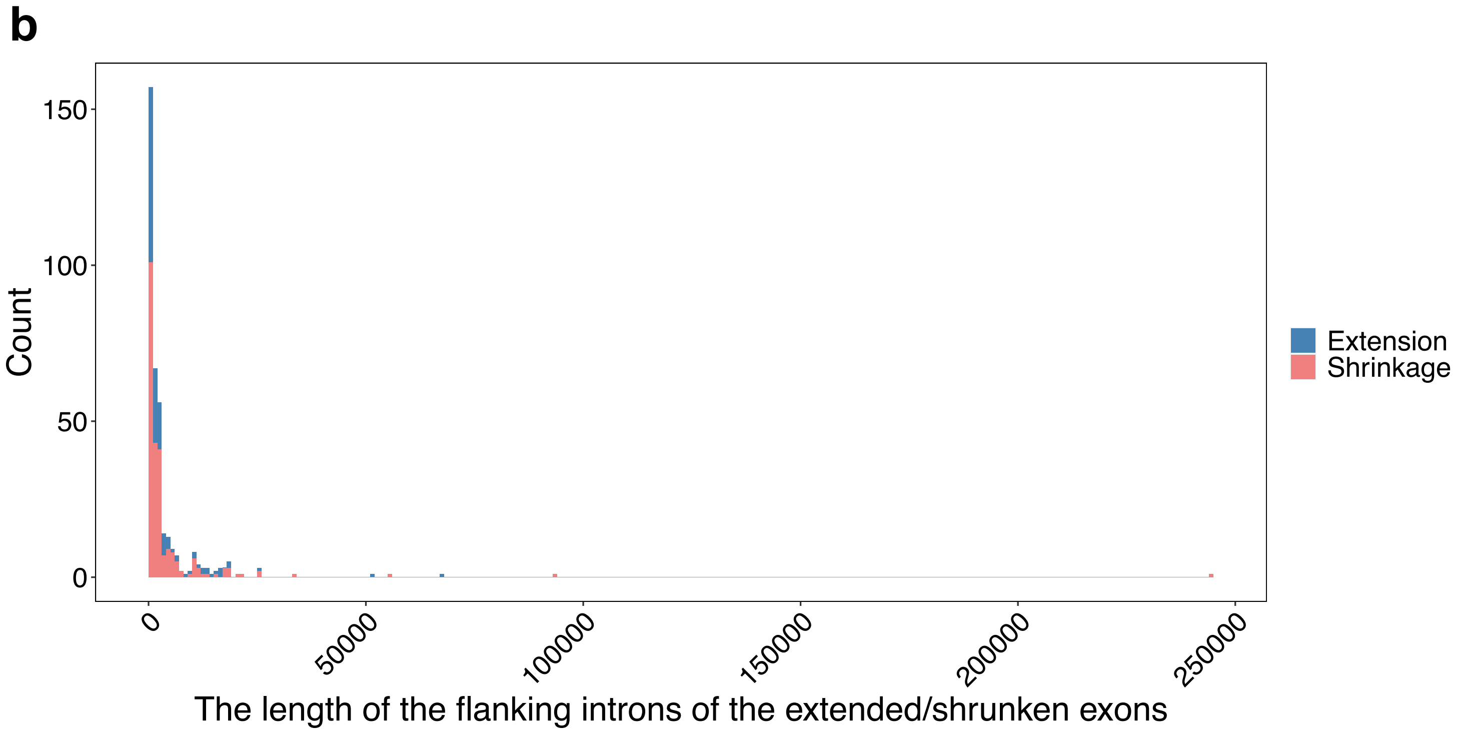

Supplemental Figure S4. (a) Histogram of the length distribution of the exons that undergo exon extension/shrinkage events. (b) Histogram of the length distribution of the flanking introns of the extended/shrunken exons.

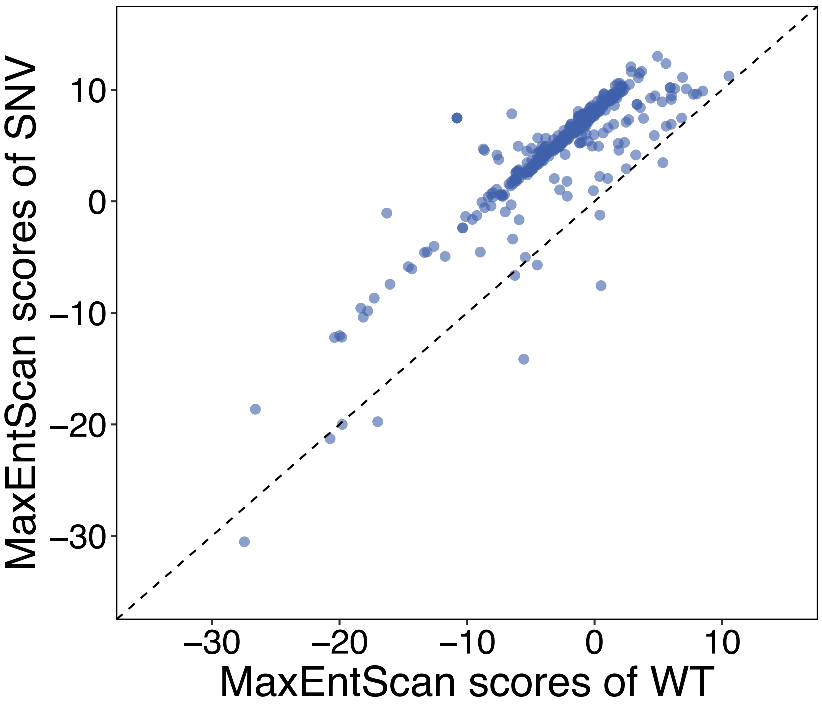

Supplemental Figure S5. Scatter plot of the strength of the splice site before and after the identified SCMs. The x- and y-axes indicate the MaxEntScan scores for the wild-type and mutated sequences, respectively.

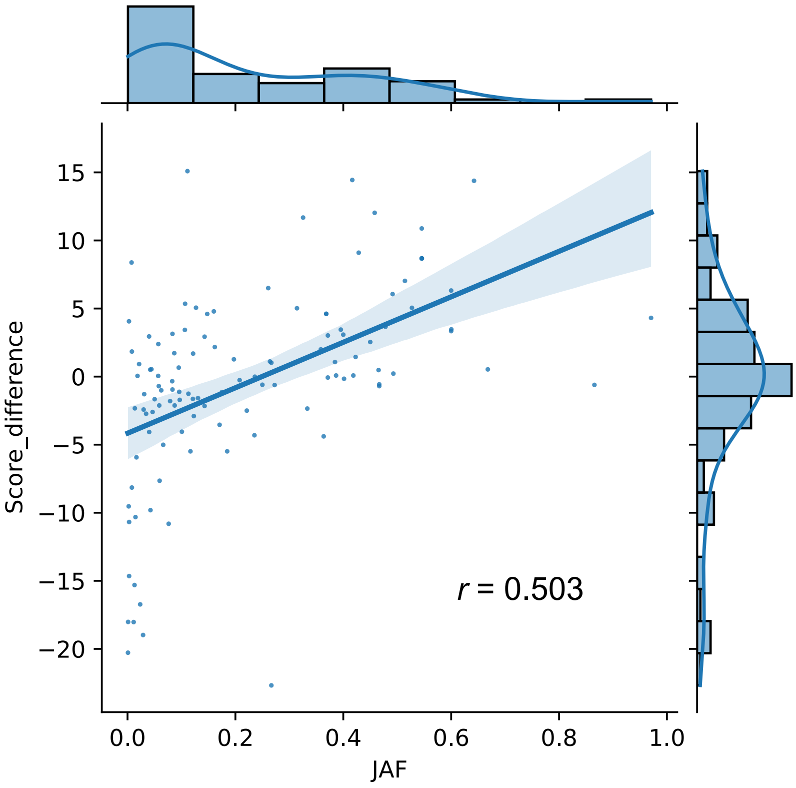

Supplemental Figure S6. Jointplot of the correlation between splice site strength and junction allele fraction. The x-axis represents the proportion of the junction reads supporting exon extension/shrinkage events. The y-axis represents the difference in MaxEntScan scores based on the splice site sequences of extended/shrunken exon and annotated exon for each exon extension/shrinkage event. Only the data points for the samples with 10 or more junction reads and heterozygous for the SCMs are used in this plot.

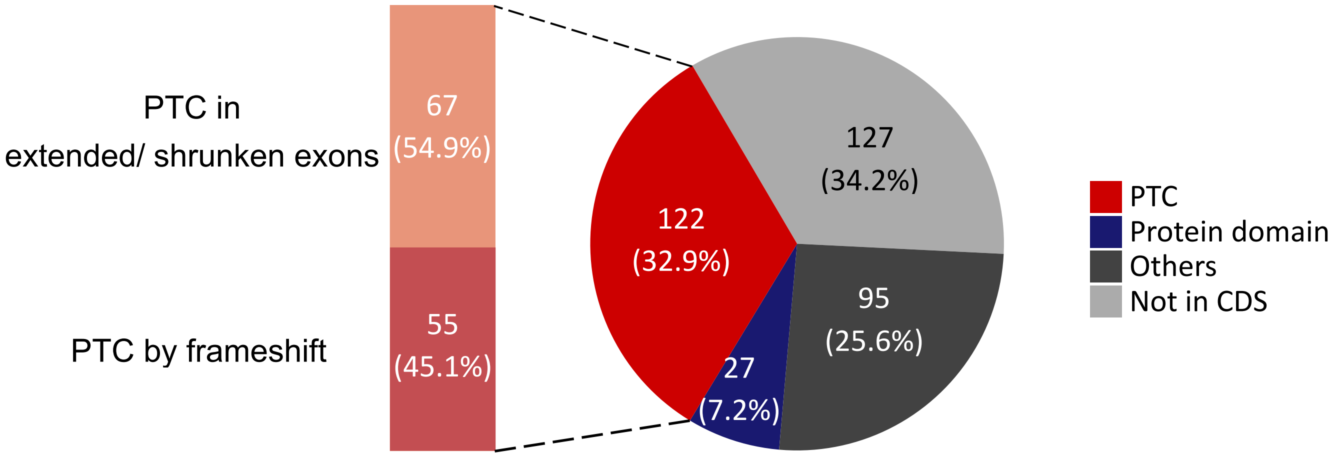

Supplemental Figure S7. Pie chart of functional effect of the extended/shrunken exons on the transcripts and proteins. "PTC" indicates the exon extension/shrinkage events that introduce PTCs, these are further divided into two categories: events that generate PTC by frameshift and events that generate PTC in extended/shrunken exons. "Protein domain" indicates the extended/shrunken exons that may disrupt the structure of the protein domain. "Not in CDS" indicates the extended/shrunken exons that are outside the protein coding region. And "Others" indicates the extended/shrunken exons that do not seem to introduce PTCs or disrupt protein domains. The numbers indicate the number of exon extension/shrinkage events.
